# Targeting skin aging: anti-senescent potential and key enzyme modulation of *Lavandula* essential oils

**DOI:** 10.3389/fphar.2026.1796715

**Published:** 2026-09-03

**Authors:** Jorge M. Alves-Silva, Joana Domingues, Fernanda Delgado, Teresa Ribeiro, Teresa Cruz, Lígia Salgueiro, Henrique Girão, Mónica Zuzarte

**Affiliations:** 1 Coimbra Institute for Clinical and Biomedical Research (iCBR), Faculty of Medicine, University of Coimbra, Coimbra, Portugal; 2 Center for Innovative Biomedicine and Biotechnology (CIBB), University of Coimbra, Coimbra, Portugal; 3 Clinical Academic Centre of Coimbra (CACC), Coimbra, Portugal; 4 Plant Biotechnology Centre of Beira Interior (CBPBI), Castelo Branco, Portugal; 5 Health Sciences Research Centre (CICS), University of Beira Interior, Covilhã, Portugal; 6 Polytechnic University of Castelo Branco-School of Agriculture (IPCB-ESA), Castelo Branco, Portugal; 7 Research Centre for Natural Resources, Environment and Society, Polytechnic University of Castelo Branco (CERNAS-IPCB), Castelo Branco, Portugal; 8 Center for Neuroscience and Cell Biology (CNC), University of Coimbra, Coimbra, Portugal; 9 Faculty of Pharmacy, University of Coimbra, Coimbra, Portugal; 10 Department of Chemical Engineering, Faculty of Sciences and Technology, Chemical Engineering and Renewable Resources for Sustainability (CERES), University of Coimbra, Coimbra, Portugal

**Keywords:** collagenase, fibroblasts, lavenders, natural products, senotherapeutic

## Abstract

**Introduction:**

Skin aging is an inherent biological process linked to chronological aging that can be accelerated by external factors. Cellular senescence and skin enzymes are crucial players in this process and, therefore, relevant preventive and therapeutic targets. In this scenario, aromatic plants and their essential oils have gained attention as promising agents in the management of skin aging. The present study aims to explore the anti-senescent potential of *Lavandula stoechas* subsp. *luisieri* and *Lavandula pedunculata* essential oils on skin fibroblasts and their effect on relevant enzymes, given their anti-inflammatory effects.

**Methods:**

The chemical characterization of the essential oils was carried out by gas chromatography-mass spectrometry and their effect evaluated on etoposide-induced senescent fibroblasts during the recovery phase. Relevant markers of cellular senescence were analyzed, namely, β-galactosidase activity through a colorimetric assay, nuclear alterations by immunofluorescence, SASP secretion through ELISA, expression levels of cell cycle inhibitors by WB analysis and ultrastructural alterations using transmission electron microscopy. Modulation of key enzyme activity was determined using an *in chemico* approach.

**Results:**

The essential oil of *L. pedunculata* was characterized by high amounts of fenchone and camphor whereas that of *L. stoechas* subsp. *luisieri* showed a very peculiar chemical profile rich in rare necrodane derivatives and lavandulyl acetate. Both essential oils were effective in decreasing most of the senescence markers but *L. stoechas* subsp. *luisieri* strikingly attained significant effects at very low concentrations (6.25 μg/mL).

**Conclusion:**

These results highlight the potential of *L. stoechas* subsp. *luisieri* to mitigate cellular senescence and delay skin aging, thereby contributing to the valorization of this underexplored species in cosmeceutical and pharmaceutical applications.

## 1 Introduction

The 2015 World Report on Aging and Health emphasized the importance of healthy aging, highlighting skin health as a vital component of overall wellbeing throughout lifespan (World Health Organization, 2015). Skin aging is a natural process associated with chronological aging that can be accelerated by several external factors, such as photoaging and cancer treatments. Its manifestations range from pigmentation alterations and loss of elasticity to impaired recovery response against damage and pathological skin disorders (Kim et al., 2022). For instance, cutaneous infections are common among the elderly due to a reduction in both the skin’s barrier function and its immune defenses. Additionally, impaired wound healing further escalates the risk of infection. It is also widely established that senescent cells accumulate in the skin with age, driving inflammaging, a chronic and low-grade inflammatory state that underlies several age-related skin disorders (Agrawal et al., 2023). Although senescent cells lose their ability to proliferate, they remain metabolically active and influence the surrounding tissues. For example, the crosstalk between senescent skin fibroblasts and melanocytes has been associated with the development of skin pigmentation (Kim et al., 2022). Furthermore, cellular senescence plays a relevant role in the loss of elasticity and wrinkle formation during aging, due to an increased activity of extracellular matrix-degrading enzymes such as elastase, collagenase and hyaluronidase (Shin et al., 2023). In light of these factors, together with longer life expectancy and rising consumer demand for anti-aging solutions, interest in this research area has increased substantially, with natural and ecofriendly cosmetic products, including plant extracts, gaining significant attention (Ribeiro et al., 2015). Indeed, a recent trend has emerged in the cosmetic industry that not only aims at improving skin aging but also promotes skin health by preventing or treating skin-related diseases (Cavinato et al., 2017). This has led to the term “cosmeceutical” which refers to cosmetic products containing active ingredients, that besides their aesthetic benefits, improve skin function and structure (Santos et al., 2019). Notably, the importance of plant extracts, including essential oils and their metabolites, in the field of skin care, has been recognized for millennia. In fact, several relevant properties have been ascribed to these compounds, namely, antioxidant, tyrosinase-inhibitory, antimicrobial, anti-inflammatory, and wound healing effects (Ribeiro et al., 2015; Cavinato et al., 2017; Santos et al., 2019).

Species from the Lamiaceae family, particularly those in the *Lavandula* genus, are highly valued for their medicinal potential and have a long history of use in traditional medicine worldwide. Lavenders have long been associated with calming, antispasmodic, antimicrobial, and anti-inflammatory properties, and have been traditionally used to manage several conditions including anxiety, insomnia, headaches, and digestive disturbances. In Mediterranean and Middle Eastern traditions, lavenders are also used in the treatment of wounds and skin disorders due to their antiseptic properties (Lis-Balchin, 2002). Consistent with these traditional uses, compounds from this genus have been shown to modulate multiple molecular targets and biological pathways, contributing to the neuroprotective, antimicrobial, and anti-inflammatory effects (Batiha et al., 2023; Allouani et al., 2025). Reflecting their interest in the medicinal/pharmaceutical fields, clinical trials have demonstrated anxiolytic potential of the oral administration of capsules containing *Lavandula angustifolia* essential oil (Dold et al., 2023). Beyond neurological applications, *Lavandula* species have been explored for dermatological and skin-related benefits. Essential oils from *L. angustifolia* and *L. stoechas* have been reported to promote wound healing and support skin health (Demir et al., 2023; Kim et al., 2025). Collectively, these studies highlight that *Lavandula* species are versatile natural products that combine traditional uses with modern therapeutic and cosmetic applications, positioning the genus as a valuable source for innovative medicines and skincare solutions.

Having this in mind, two lavender species, *Lavandula stoechas* subsp. *luisieri* (Rozeira) Rozeira and *Lavandula pedunculata* (Mill.) Cav. were selected for this study. These species are widely distributed in Portugal but remain underexplored and underutilized. Both have been extensively reported for their anti-inflammatory and antimicrobial properties (Roller et al., 2009; Zuzarte et al., 2012; 2022; Baptista et al., 2015; Rufino et al., 2015; Arantes et al., 2016; Pombal et al., 2016; Dias et al., 2017; Nunes et al., 2017; Costa et al., 2018; Giménez-Rota et al., 2019; Miguel et al., 2020), which are highly relevant for the prevention and management of skin aging and associated conditions. In addition to the reported effects, the selected essential oils have a pleasant aroma, making them attractive candidates for further exploration in cosmetic and pharmaceutical applications.

Therefore, the present study aims to shed light on the potential of the essential oils obtained from these two species on skin aging. A special focus was placed on cellular senescence given its central role in the onset of age-related skin diseases and overall loss of skin health. Cellular senescence contributes to the dysregulation and overactivation of key skin enzymes, including tyrosinase, collagenase, elastase, and hyaluronidase, which are critically involved in skin aging processes. Specifically, collagenase degrades collagen, compromising skin structure; elastase breaks down elastin, reducing skin elasticity; hyaluronidase decreases hyaluronic acid levels, lowering skin hydration; and tyrosinase regulates melanin synthesis, contributing to pigmentation changes (Csekes and Račková, 2021). Given their pivotal roles in the maintenance of skin integrity, these enzymes were selected to evaluate whether the essential oils could directly improve skin health by modulating enzyme activity, or indirectly by preventing or delaying fibroblast senescence.

Overall, our results provide new insights into the field of skincare, paving the way for the development of innovative natural approaches to delay skin aging and improve overall skin health.

## 2 Materials and methods

### 2.1 Plant material

The aerial parts of *L. stoechas* subsp. *luisieri* (Rozeira) Rozeira and *L. pedunculata* (Mill.) Cav. (Lamiaceae) were collected in Serra da Malcata (558 m, 401206.741 N; 70622.085 W), Portugal. A voucher specimen of each species is deposited at the Herbarium of the Biology Laboratory of Polytechnic University of Castelo Branco - Agrarian School (IPCB-ESA), with the accession numbers ESACB-CBPBI-LL-M-0801 and ESACB-CBPBI-LP-M-0401, for *L. stoechas* subsp. *luisieri* and *L. pedunculata*, respectively. Plants were collected and identified by Prof. Fernanda Delgado (IPCB-ESA). Air-dried plants were hydrodistilled for 2 h in a Clevenger-type apparatus according to the procedure described in the European Pharmacopoeia (Council of Europe, 2010). The Consensus statement on the Phytochemical Characterisation of Medicinal Plant extracts (ConPhyMP) guidelines was used to ensure compliance with the best practices for the characterization of the selected natural extracts, thus ensuring reproducibility and accurate interpretations among studies with natural extracts (Heinrich et al., 2022; Society for Medicinal Plants and Natural Product Research, 2022).

### 2.2 Gas chromatography-mass spectrometry

The volatile profiles of the essential oils were obtained by gas chromatography coupled with mass spectrometry (GC/MS SCION-SQ 456 GC, Bruker Corporation, Massachusetts, United States of America). The separation was achieved on an HP-5MS capillary column (30 m × 0.25 mm id × 0.25 m film thickness, Agilent J&W, Folsom, CA, United States). Helium was the carrier gas used with a flow rate of 1 mL/min. The essential oil samples were injected with a volume of 1 μL, using a split ratio of 1:100, and analyzed using electron impact ionization mass spectrometry (EI-MS) at 70 eV. The compounds were identified in scan mode with the positive polarity of ions 20–300 m/z with a time of 250.0 ms. The initial oven temperature was programmed to 45 °C, gradually increasing 3 °C/min to 175 °C and 300 °C with a heating rate of 15 °C/min, and maintaining this temperature for 10 min. The transfer line and the ion source were programmed at 250 °C and 220 °C, respectively. Compounds were identified based on their GC retention indices and mass spectra. Retention indices, calculated by linear interpolation relative to the retention times of C7–C30 n-alkanes, were compared with those of authentic compounds, as well as with our laboratory database and literature data. Acquired mass spectra were compared with reference spectra from the laboratory database, the NIST MS library (NIST 17, version 2.3), and literature data. The relative amount of each compound was expressed as a percentage of the relative peak area of the compound, relative to the total area of the peaks identified in the samples.

### 2.3 Cell culture

The cell line NIH/3T3 (mouse embryonic fibroblast, ATCC CRL-1658) was obtained from the American Type Culture Collection and cultured in Dulbecco’s Modified Eagle’s Medium (DMEM, Gibco, Thermo Fisher Scientific, Waltham, MA, United States) with 10% fetal bovine serum (FBS, Biowest, Nuaillé, France) and 1% Penicillin/Streptomycin (Gibco) at 37 °C in a humidified incubator with 5% CO_2_. Cells were subcultured when they reached approximately 75%–80% confluency and cell morphology was checked under a light microscope. Cells between passages 15 and 20 were used for all the experiments.

### 2.4 Cell viability

The effect of different concentrations of the essential oils on fibroblasts viability was evaluated through the resazurin reduction assay, as previously reported (Piras et al., 2022). Briefly, NIH/3T3 fibroblasts (50,000 cells/mL) were seeded in 48-well plates. After an overnight stabilization, different concentrations of *L. pedunculata* (800–25 μg/mL) or *L. stoechas* subsp. *luisieri* (200–3.125 μg/mL) essential oils were added for 24 h. At the end of the experiment, the medium was removed and fresh medium containing resazurin (50 µM) was added for 2 h. The absorbance was read at 570 nm with a reference filter of 620 nm in an automated plate reader (SLT, Austria). Cell viability was determined using the following equation:

$$\text{Cell viability }\left(\%\right)=\left({\text{Abs}}_{\mathrm{Exp}}/{\text{Abs}}_{\text{CT}}\right)\times 100,$$

being ${\text{Abs}}_{\mathrm{Exp}}$ the absorbance (difference between 570 and 620 nm) in the different experimental conditions and ${\text{Abs}}_{\text{CT}}$ , the absorbance in control cells (no essential oil added).

### 2.5 Cellular senescence

#### 2.5.1 Senescence induction

Cellular senescence was induced on fibroblasts using etoposide, a topoisomerase II inhibitor. Briefly, cells were plated at the indicated cell density and left to adhere overnight. Then, etoposide (12.5 μM; Sigma-Aldrich, St. Louis, MO, United States) was added for 24 h. After this time, the medium was removed, fresh medium was added, and cells were cultured for an additional period of 24 h or 7 days, for recovery time selection. After the selection of the recovery period, following etoposide treatment, fresh medium with or without the essential oils, was added during the recovery phase.

#### 2.5.2 Senescence-associated β-galactosidase activity

Cells were plated at 15,000 and 30,000 cells/mL (for 24 h recovery period) or 1,500 and 20,000 cells/mL (for a 7-day recovery period), for control or etoposide-treated cells, respectively. Following this period, in the presence or absence of the essential oils (200 or 100 μg/mL for *L. pedunculata* and 6.25 or 3.125 μg/mL for *L. stoechas* subsp. *luisieri*), senescence associated (SA)-β-galactosidase activity was determined using a commercially available kit, according to the manufacturer’s protocol (#9860, Cell Signaling Technology, Inc., Danvers, MA, United States). Cells were imaged using a brightfield microscope (VWR) for quantitative analysis using ImageJ software v1.54p with senescent cells presenting the characteristic blue stain, indicative of β-galactosidase activity. Results were presented as a percentage of SA-β-gal-positive cells, with a minimum of 100 cells being imaged in each independent experiment.

#### 2.5.3 γH2AX and Ki67 immunostaining

Following cell plating and treatments as described in Section 2.5.2, after 7 days, cells were fixed with 4% paraformaldehyde for 10 min and subsequently washed with PBS. Then, cells were permeabilized with 0.1% Triton X-100 for 15 min, washed with PBS and blocked with 3% bovine serum albumin and 10% goat serum in PBS for 1 h. Proteins of interest were detected using a primary antibody against the phosphorylated form of the histone variant H2AX - γH2AX (1:500, #9718 Cell Signaling) or Ki67 (1:100, sc-23900 Santa Cruz Biotechnology), applied to cells overnight at 4 °C. Afterward, cells were washed three times with PBS and incubated for 1 h, at room temperature, with the respective secondary antibody (1:500, goat anti-rabbit Alexa Fluor 568 or 1:500 goat anti-mouse Alexa Fluor 633) and DAPI for nuclear staining (1:1,000). Following washes with PBS, coverslips were mounted on glass slides with Mowiol mounting medium. Images were obtained using a confocal point-scanning microscope (Zeiss LSM710; Carl Zeiss, Germany) with a ×63 objective, for γH2AX staining or a ×40 objective for Ki67 analysis. The number of puncta per nucleus was determined using the Yen threshold setting with manual adjustment of ImageJ v1.54p and the “analyse particles” function of ImageJ. The nuclear area was determined using DAPI staining as input image on CellProfiler v4.2.8. Ki67 was counted manually and expressed as a percentage of Ki67-positive cells per total cells.

#### 2.5.4 p21 and p53 expression

Cells were seeded at 5,000 or 100,000 cells/mL (for control or etoposide-treated cells, respectively) and treated as described in Section 2.5.2. Following 7 days, cells were lysed with RIPA buffer freshly supplemented with 1 mM dithiothreitol, protease and phosphatase inhibitor cocktails (Sigma-Aldrich, St. Louis, MO, United States) and left on ice for 30 min before vigorous vortexing for 5 s. The nuclei and the insoluble cell debris were removed by centrifugation at 4 °C, at 12,000 *g* for 10 min and the supernatants were used as total cell lysates. Protein concentration was determined by the bicinchoninic acid protein assay (Thermo Fisher Scientific) and cell lysates were denaturated in sample buffer (0.125 mM Tris pH 6.8, 2% (w/v) sodium dodecyl sulfate (SDS), 100 mM dithiothreitol (DTT), 10% glycerol and bromophenol blue). For protein separation, samples (35 µg/lane) were electrophoretically separated in a 12% (v/v) SDS-polyacrylamide gel at 130 V, during 1.5 h, at room temperature. Protein lines were then transferred, during 3 h at 400 mA, using a refrigerated (4 °C) wet transference system, to polyvinylidene fluoride membranes (Millipore, Burlington, MA, United States), which were previously activated with methanol. The membranes were incubated for 1 h at room temperature with 5% (w/v) skim milk in tris-buffered saline with Tween 20 (TBS-T). Then, an overnight incubation at 4 °C with antibodies against p21 (1:1,000, Abcam ab188224) or p53 (1:1,000, Proteintech 10442-1-AP) was carried out. Finally, the membranes were washed for a total of 30 min with TBS-T and incubated for 1 h at room temperature with horseradish peroxidase-conjugated secondary antibody (1:10,000; #7074, Cell Signaling Technology). An anti-tubulin antibody (1:20,000; T7816, Sigma-Aldrich) was used as a loading control. The immunocomplexes were detected using a chemiluminescence scanner (Image Quant LAS 500, GE) and protein quantification performed on ImageLab software version 6.1.0 (Bio-Rad Laboratories Inc.). Protein levels were normalized to both tubulin and etoposide-treated cells alone.

#### 2.5.5 Senescence-associated secretory phenotype

Cells were seeded at 5,000 or 100,000 cells/mL (for control or etoposide-treated cells, respectively) and treated as described in Section 2.5.2. Following 7 days, cell culture supernatants were collected and centrifuged for 20 min, at 1,000 *g* at 4 °C. Cells were collected in RIPA buffer freshly supplemented with 1 mM dithiothreitol, protease and phosphatase inhibitor cocktails and left on ice for 30 min before vigorous vortexing for 5 s. The nuclei and the insoluble cell debris were removed by centrifugation at 4 °C, at 12,000 *g* for 10 min and the supernatants were used as total cell lysates. Protein concentration was determined by the bicinchoninic acid protein assay (BCA, Thermo Fisher Scientific). Cell culture supernatants were then used to determine secreted IL-1β (RTFI00904, Assay Genie, Dublin, Ireland), IL-6 (RTFI00034, Assay Genie), MMP3 (TRFI00968, Assay Genie) and TNF-α (RTFI01177, Assay Genie) using an ELISA kit, according to manufacturer’s instructions. The amount of secreted protein was normalized to the amount of total cell protein. At least three independent experiments were performed.

#### 2.5.6 Cell ultrastructure

Fibroblasts seeded at 5,000 or 100,000 cells/mL (for control and etoposide-treated cells, respectively) were treated as described in Section 2.5.2. Cells were briefly fixed with 2.5% glutaraldehyde (v/v) prepared in 0.1 M sodium cacodylate buffer, pH 7.2, directly in the culture wells containing medium, prior to collection by scraping. The resulting cell pellets were fixed for additional 2 h. Following rinsing in the same buffer, post-fixation was performed using 1% osmium tetroxide for 1 h. After rinsing in buffer, buffer and distilled water and a final rinsing step in distilled water, 1% aqueous uranyl acetate was added to the cells for contrast enhancement during 1 h. Following embedding in 2% molten agar, cell pellets were dehydrated through a graded acetone series (30%–100%), impregnated and included in Epoxy resin (Sigma). Resin blocks were polymerized at 60 °C for 48 h and ultrathin sections (∼70 nm) obtained with an ultramicrotome (Leica UM6), mounted on copper grids (TAAB Laboratories) and stained with 2% uranyl acetate and 3% lead citrate. Observations were carried out on a FEI Tecnai G2 Spirit BioTwin electron microscope at 100 kV.

### 2.6 *In vitro* enzyme activities

#### 2.6.1 Assessment of collagenase activity

The activity of collagenase was determined using a previously described method (Jiratchayamaethasakul et al., 2020). Briefly, 1 mg of Azo dye-impregnated collagen was added to individual microcentrifuge tubes followed by 800 μL of 0.1 M Tris-HCl, pH 7. Then, 100 μL of the essential oils (800 and 400 μg/mL) was added to the tubes. The tubes were then randomly divided into two groups, one with and another without the enzyme. To the former, 100 μL of collagenase (200 U/mL) was added and to the latter 100 μL of the buffer was added. The tubes were then incubated for 1 h at 43 °C, followed by a centrifugation at 3,000 rpm for 10 min to pellet undegraded collagen. The supernatant (200 μL) was transferred to a 96-well plate, and the absorbance was measured at 550 nm. Blank reaction was performed by adding 100 μL of the buffer instead of the essential oil. Collagen activity was determined as follows:

$$\text{Collagen activity }\left(\%\right)=\left[\frac{C-\left(\text{Sx}-\text{Sxb}\right)}{C}\right]\times 100,$$

being C the absorbance of the blank tube, Sx the absorbance of the sample with enzyme and Sxb is the absorbance of the sample without enzyme.

#### 2.6.2 Assessment of tyrosinase activity

Tyrosinase activity was assessed by the method previously reported (Jiratchayamaethasakul et al., 2020) with slight modifications. In brief, to 110 μL of sodium phosphate buffer (0.1 M), pH 6.8, 10 μL of the essential oils (800–12.5 μg/mL) and 10 μL of tyrosinase enzyme solution (1,500 U/mL) were added and the solution was incubated for 10 min at 37 °C. As a control, the tyrosinase solution was replaced by the buffer. After the incubation step, 20 μL of L-tyrosine (1.5 mM) in phosphate buffer, pH 6.8, was added. Following an additional incubation for 30 min at 37 °C, the absorbance was read at 490 nm. A black reaction was carried out by replacing the essential oil with 10 μL of the buffer. Tyrosinase activity was determined as follows:

$$\text{Tyrosinase activity }\left(\%\right)=\left[\frac{C-D}{A-B}\right]\times 100,$$

being A and B the absorbance of the blank with and without tyrosinase, respectively, and C and D the absorbance of the sample with and without tyrosinase.

#### 2.6.3 Assessment of hyaluronidase activity

Hyaluronidase activity was determined as follows: to a microcentrifuge tube, 10 μL of hyaluronidase (8 mg/mL in 0.1 M acetate buffer, pH 3.6) was mixed with 10 μL of the essential oils (800–200 μg/mL) and incubated for 20 min at 37 °C. Then, 20 μL of calcium chloride (12.5 mM) was added and incubated for 20 min at 37 °C. Next, 50 μL of hyaluronic acid (1.2 mg/mL in acetate buffer) was added and the solution was incubated for 40 min at 37 °C. To inactivate hyaluronidase, 2 μL of NaOH (0.4 N) and 20 μL of potassium tetraborate (0.4 N) were added to the tubes and incubated for 3 min at 100 °C. Finally, 600 μL of (dimethylamino) benzaldehyde (0.01 M in glacial acetic acid made from a stock solution of 0.08 M in 5:1 mixture of glacial acetic acid:hydrochloric acid 10 N) was added for 20 min at 37 °C until pink color development. Then, 200 μL of this solution was transferred to a 96-well plate and the absorbance was read at 585 nm. Blank conditions were achieved by adding 10 μL of acetate buffer instead of the essential oil. Hyaluronidase activity was determined using the following equation:

$$\text{Hyaluronidase activity }\left(\%\right)=\left[\frac{\text{Ct}-S}{\text{Ct}}\right]\times 100,$$

being Ct the absorbance of the blank and S the absorbance of the essential oil.

#### 2.6.4 Assessment of elastase activity

Elastase activity inhibition was assessed according to the method reported by Jiratchayamaethasakul et al. (2020), with slight modifications. Briefly, 100 μL of Tris-HCl (0.2 M), pH 8.0, was mixed with 25 μL of N-succinyl-Ala-Ala-Ala-p-nitroanilide (10 mM) and 50 μL of various concentrations of the essential oils (800–12.5 μg/mL) and left to incubate for 15 min at 25 °C. Prior to the addition of 25 μL of elastase enzyme solution (0.3 U/mL), the absorbance was measured (B and D), and after enzyme addition, the tubes were incubated for 15 min at 25 °C. A blank reaction was performed with 50 μL of the buffer being added instead of the essential oil. The absorbance was measured at 410 nm, and elastase activity was determined using the following equation:

$$\text{Elastase activity }\left(\%\right)=\left[\frac{C-D}{A-B}\right]\times 100,$$

being A and B, the absorbance of the blank after and before incubation with the enzyme, respectively, and C and D, the absorbance of the sample after and before incubation with the enzyme.

### 2.7 Statistical analysis

Results for biological assays are shown as mean ± standard error the mean (SEM) of at least three independent experiments represented by bars, with individual points shown as scattered dots. For cell viability, ELISA and Western blot analysis results were normalized to control or etoposide conditions, respectively. Distribution normality was assessed using Shapiro-Wilk or Kolmogorov normality tests. When sample normality was confirmed, statistical significance was determined using one-way or two-way ANOVA or mixed-effects analysis followed by Tukey’s, Dunnett’s, Holm-Šídák’s or Šídák’s multiple comparison test. In case of non-normality, Kruskal–Wallis followed by Dunn’s multiple comparison test was used.

## 3 Results

### 3.1 Chemical composition of *Lavandula* spp. essential oils

Several samples of *L. pedunculata* and *L. stoechas* subsp. *luisieri* essential oils were analysed. A detailed chemical profile of the most representative samples found in Serra da Malcata region is presented in Table 1, these being selected to pursue further experiments. Both essential oils were characterized by high amounts of oxygenated monoterpenes with *L. pedunculata* being particularly rich in fenchone (63.8%) and camphor (20%). Regarding *L. stoechas* subsp. *luisieri*, a very peculiar chemical profile was found as necrodane derivatives, very rare compounds in the plant kingdom, attained 28.1% of the total composition with trans-α-necrodyl acetate (19.1%) being the most predominant. In addition, lavandulyl acetate (13.6%) and camphor (8.8%) were present in relatively high amounts (Table 1). In addition, non-identified compounds above 1% were also included and their respective mass spectra included as supplementary material.

**TABLE 1:** Chemical profile of a representative sample of *Lavandula stoechas* subsp. *luisieri* and of *Lavandula pedunculata* from Serra da Malcata, Portugal.

Identified compound	R.T. (min.)	RI_exp_	RI_lit_	Percentage in the samples (%)	
				*L. stoechas* subsp*. luisieri*	*L. pedunculata*
Tricyclene	7.4	910	914	—	0.1 ± 0.0
α-Pinene	7.8	920	924	2.3 ± 0.1	4.8 ± 0.0
3,5-Dimethylene-1,4,4-trimethylcyclopentene	7.9	922	924	3.1 ± 0.0	—
Camphene	8.4	934	933	0.5 ± 0.0	1.9 ± 0.0
2,4(10)-Thujadiene	8.6	950	955	0.2 ± 0.0	—
1,2,3,3-Tetramethyl-cyclopenten-4-one	11.2	1,003	1,009	0.4 ± 0.0	—
*p*-Cymene	11.5	1,011	1,010	0.2 ± 0.0	0.3 ± 0.0
Limonene	11.7	1,015	1,014	—	1.8 ± 0.0
1,8-Cineole	11.8	1,024	1,028	6.1 ± 0.1	1.9 ± 0.0
*cis*-Linalool oxide (furanoid)	13.7	1,063	1,065	0.7 ± 0.0	—
1,3,4,4-Trimethyl-2-cyclohexen-1-one	14.0	1,071	1,077	1.2 ± 0.0	—
Fenchone	14.3	1,080	1,080	3.2 ± 0.1	**63.8 ± 0.0**
*trans*-Linalool oxide (furanoid)	14.4	1,081	1,083	0.4 ± 0.0	—
Linalool	15.0	1,095	1,099	2.1 ± 0.1	0.5 ± 0.0
Fenchol	15.5	1,108	1,112	-	0.8 ± 0.0
Camphor	16.9	1,142	1,148	8.8 ± 0.1	**20.0 ± 0.1**
*trans*-α-Necrodol	17.1	1,147	1,151	5.3 ± 0.2	—
*endo*-Borneol	17.9	1,167	1,166	—	0.3 ± 0.0
n.d. 1	18.0	1,171	​	2.9 ± 0.1	0.1 ± 0.0
*cis*-α-Necrodol	18.3	1,178	1,182	1.9 ± 0.1	—
*p*-Cymen-8-ol	18.9	1,191	1,188	—	0.2 ± 0.0
5-Methylene-2,3,4,4-tetramethylcyclopent-2-enone	19.0	1,194	1,198	5.5 ± 0.1	—
α-Terpineol	19.1	1,199	1,196	—	0.2 ± 0.0
Verbenone	19.9	1,210	1,206	0,7 ± 0,1	0.3 ± 0.0
Fenchyl acetate	20.4	1,227	1,228	—	0.4 ± 0.0
Bornyl acetate	23.2	1,266	1,284	​	1.9 ± 0.0
n.d. 2	23.4	1,297	​	1.9 ± 0.0	—
*trans*-α-Necrodyl acetate	23.4	1,302	1,296	**19.1 ± 0.4**	—
Lavandulyl acetate	23.7	1,310	1,312	**13.6 ± 0.1**	—
*cis*-α-Necrodyl acetate	24.2	1,322	1,324	1.8 ± 0.0	—
α-Cadinene	33.3	1,546	1,541	1.0 ± 0.0	0.2 ± 0.00
Caryophyllene oxide	35.6	1,595	1,589	0.4 ± 0.0	—
n.d. 3	35.9	1,604	​	5.0 ± 0.2	—
Globulol	36.4	1,621	1,591	2.6 ± 0.1	—
τ-Cadinol	37.8	1,657	1,648	0.5 ± 0.0	—
Total identified compounds (%)		​	​	**81.7**	**99.5**

Compounds expressed as a percentage (%) of peak area ± standard deviation (n = 2) and listed in order of elution on the HP-5MS column. RI_exp_: experimental retention index calculated relative to n-alkanes series standards (C7–C30) on the HP-5MS column. RI_lit_: literature retention index. Major compounds are presented in bold. n.d., non identified compounds in the studied samples; R.T., retention time of the eluted compounds expressed in minutes.

### 3.2 Safety profile and proliferative effects of *Lavandula* spp. essential oils

**FIGURE 1:**
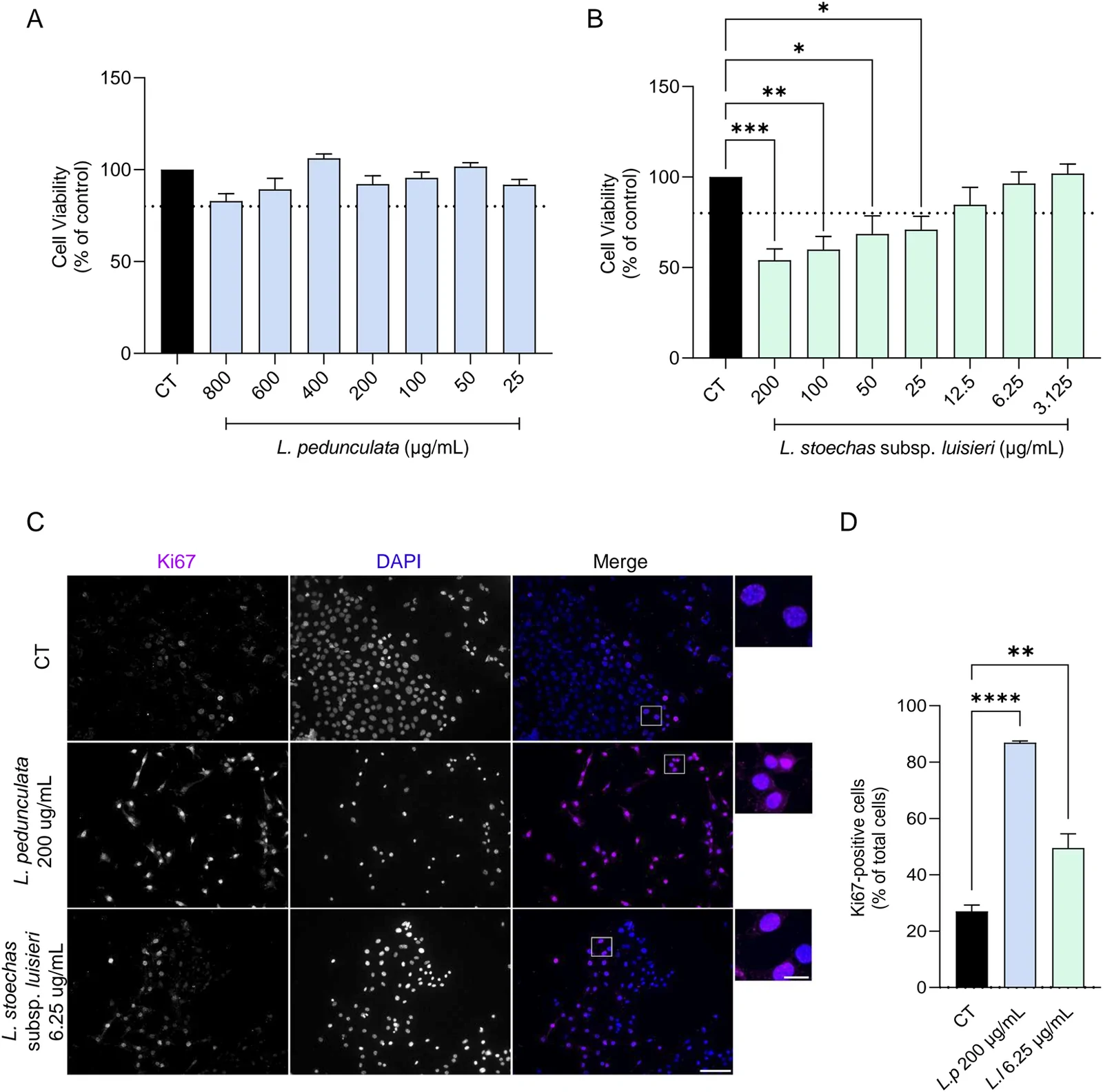
Effect of *Lavandula pedunculata* and *Lavandula stoechas* subsp. *luisieri* essential oils on cell viability and proliferation. **(A)** Effect of *Lavandula pedunculata* essential oil on NIH/3T3 viability. **(B)** Effect of *L. stoechas* subsp. *luisieri* essential oil on NIH/3T3 viability. **(C)** Representative fluorescence images of NIH/3T3 cells treated for 7 days with *Lavandula pedunculata* and *Lavandula stoechas* subsp. *luisieri* essential oil and then stained with the proliferation marker Ki67 (Alexa Fluor 647). Scale bars: 100 μm; 50 µm (Zoom-ins). **(D)** Percentage of Ki67-positive cells. Results show the mean ± SEM (n = 3–4). *p < 0.05, **p < 0.01, ***p < 0.001, ****p < 0.0001, compared to control (CT) cells, assessed by Kruskal–Wallis followed by Dunn’s multiple comparison test (for **A**) or one-way ANOVA followed by Dunnett’s multiple comparison test (for **B**, **D**).

The safety profile of the selected essential oils was evaluated using NIH/3T3 fibroblasts to determine the non-toxic concentrations suitable for bioactivity experiments. As observed in Figure 1, *L. pedunculata* essential oil was safe even at the highest concentration tested (800 μg/mL; Figure 1A) while *L. stoechas* subsp. *luisieri* essential oil decreased cell viability. For the latter, statistical significance was observed at 25 μg/mL and treatment with 12.5 μg/mL maintained approximately 80% of cell viability, generally regarded as the threshold for non-cytotoxicity (Figure 1B). Bearing this in mind, for the following experiments, two intermediate concentrations of *L. pedunculata* (200 μg/mL and 100 μg/mL) essential oil were used while for *L. stoechas* subsp. *luisieri* much lower concentrations were selected (6.25 μg/mL and 3.125 μg/mL). Interestingly, at the highest concentrations (200 μg/mL for *L. pedunculata* and 6.25 μg/mL for *L. stoechas* subsp. *luisieri*), the essential oils increased the number of Ki67-positive cells (Figure 1C), a cellular proliferation marker, suggesting a positive effect on cell proliferation after 7 days, although a lower statistical significance was attained for *L. stoechas* subsp. *luisieri* (Figure 1D).

### 3.3 Recovery time selection for cellular senescence experiments

**FIGURE 2:**
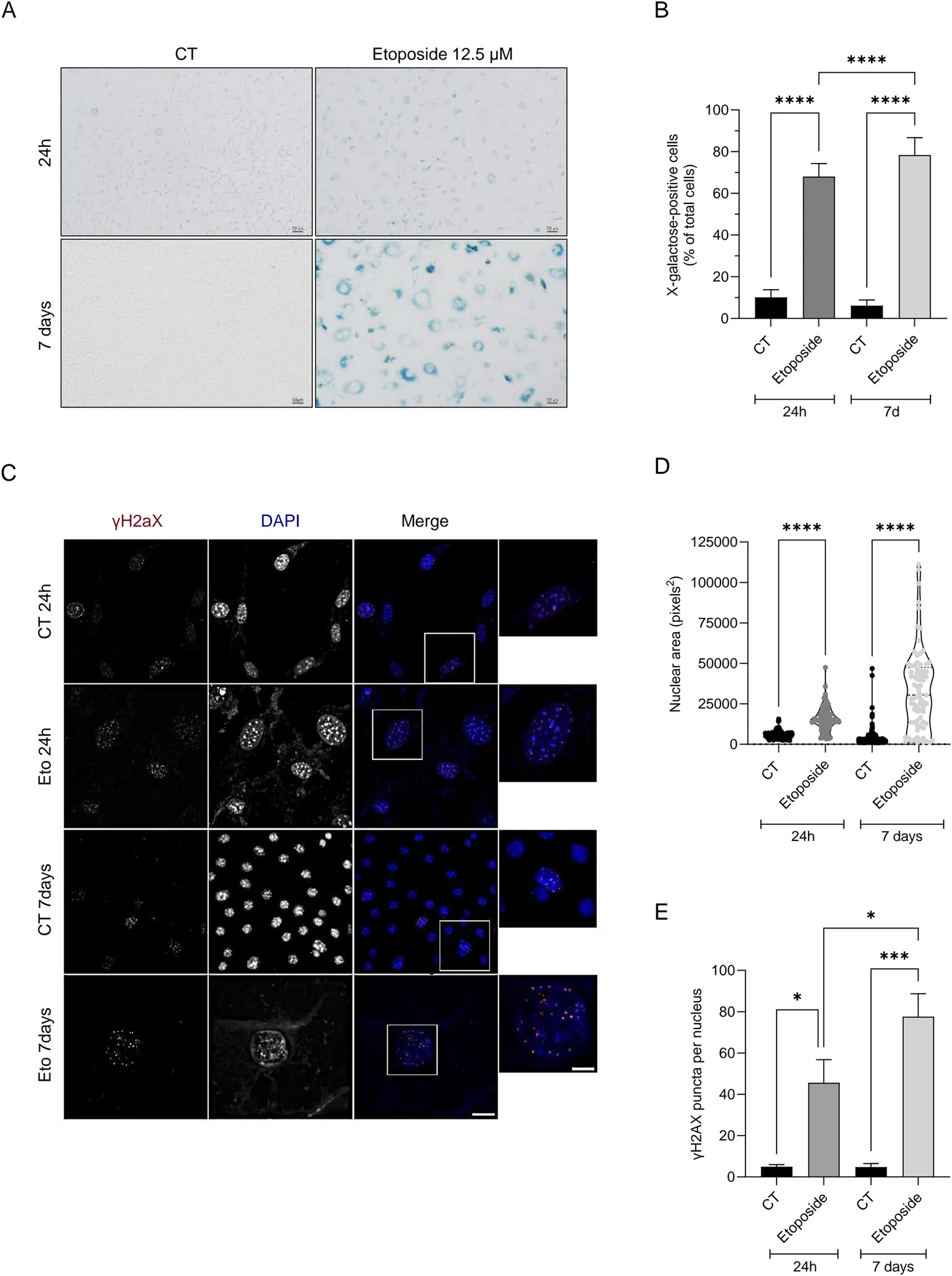
Effect of recovery time on senescence induction. **(A)** Representative brightfield images of NIH/3T3 cells treated with etoposide for 24 h followed by 24 h or 7 days of recovery in etoposide-free medium. Senescent cells presented a distinct blue color. **(B)** Percentage of Senescence associated-β-galactosidase-positive cells. **(C)** Representative confocal images of NIH/3T3 cells treated with etoposide for 24 h followed by 24 h or 7 days of recovery in etoposide-free medium, followed by immunofluorescence for γH2AX (Alexa Fluor 647). Scale bar: 100 nm, 50 nm (Zoom-ins). **(D)** Nuclear areas shown as pixels^2^. **(E)** γH2AX puncta per nucleus. Results show the mean ± SEM (n = 3, minimum of 100 cells/n). *p < 0.05, ***p < 0.001 and ****p < 0.0001, compared to control (CT) or etoposide-treated (Eto) cells, using one-way ANOVA followed by Tukey’s (for **B**) or Dunnett’s multiple comparison test (for **E**) and Kruskal–Wallis analysis followed by Dunn’s multiple comparison test (for **D**).

In order to understand if NIH/3T3 cells have a time-dependent response to etoposide stimulus, several cellular senescence markers were assessed, at two divergent time-points of cellular recovery, namely, 24 h and 7 days. Following 24 h of etoposide treatment, fibroblasts showed a significant increase in typical senescence markers such as a positive stain for senescence-associated β-galactosidase (SA-β-gal; Figures 2A,B), an increased nuclear area (Figures 2C,D) and a significant accumulation of the phosphorylated form of the histone γH2AX (Figures 2C,E), despite presenting a control-like morphology (Figure 2A). Strikingly, following 7 days of recovery, senescent features were markedly pronounced. SA-β-gal staining was more obvious, and cells were visibly larger (Figures 2A,B). Corroborating this finding, nuclear area was increased, and nucleus had a more significant accumulation of γH2AX, suggesting an accumulation of double strand breaks that failed to repair. Considering these results, the following experiments were conducted using the recovery period of 7 days.

### 3.4 Anti-senescent potential of *Lavandula* spp. essential oils

#### 3.4.1 Effect on cell proliferation

**FIGURE 3:**
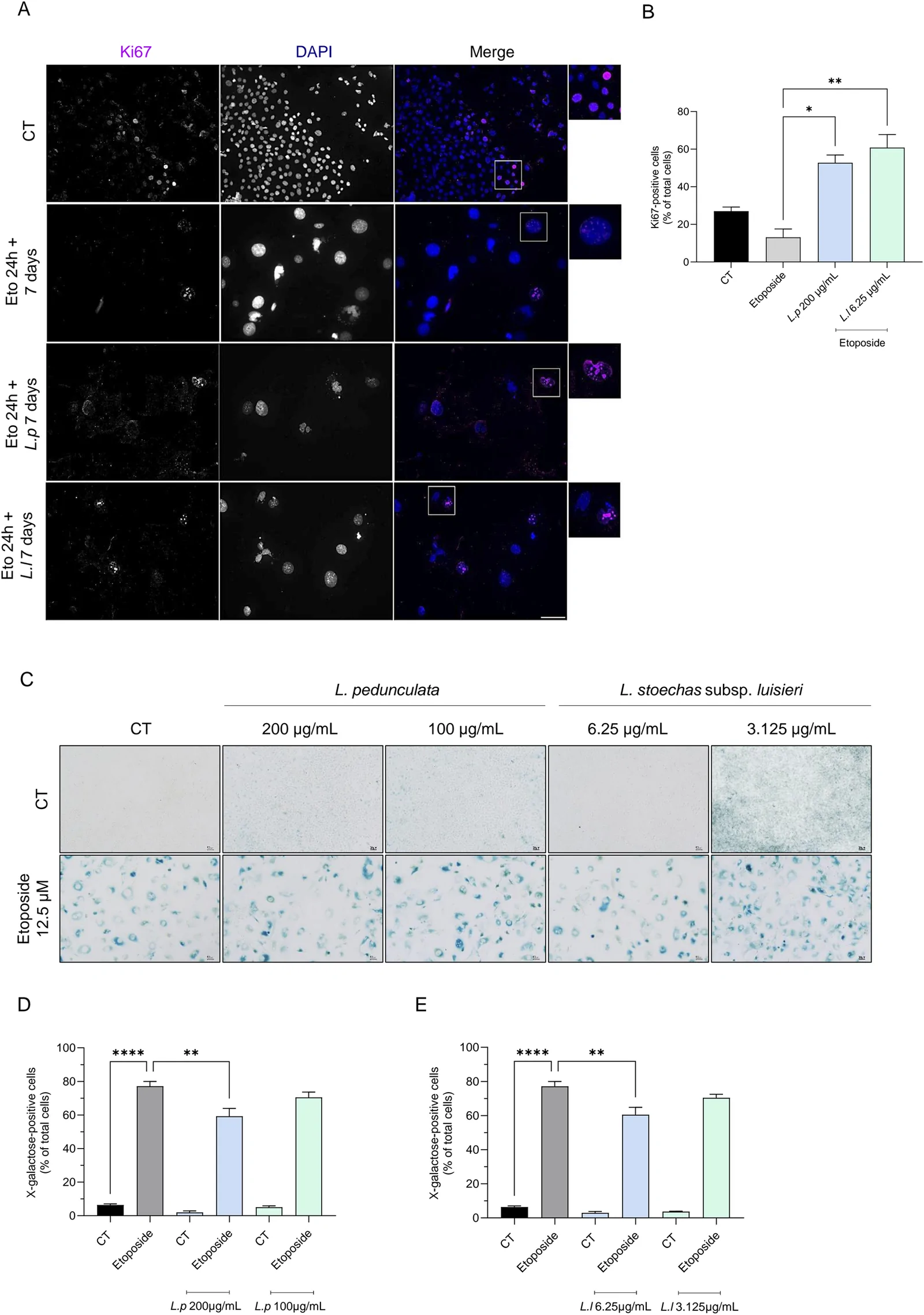
Effect of *Lavandula pedunculata* and *Lavandula stoechas* subsp. *luisieri* essential oils on cell proliferation and etoposide-induced β-galactosidase activity. **(A)** Representative fluorescence images of NIH/3T3 cells treated with etoposide for 24 h followed 7 days of recovery in etoposide-free medium in the presence or absence of the essential oils, followed by immunofluorescence for Ki67 (Alexa Fluor 647). Scale bar: 100 nm, 50 nm (Zoom-ins). **(B)** Percentage of Ki67-positive cells. **(C)** Representative brightfield images of NIH/3T3 cells treated with etoposide for 24 h followed by 7 days of recovery in etoposide-free medium in the presence or absence of the essential oils. Senescent cells presented a distinct blue color. **(D)** Percentage of Senescence associated (SA)-β-galactosidase-positive cells in the presence of *Lavandula pedunculata* essential oil. **(E)** Percentage of SA-β-galactosidase-positive cells in the presence of *Lavandula stoechas* subsp. *luisieri* essential oil. Results show the mean ± SEM (n = 3–5, minimum 100 cells/n). *p < 0.05, **p < 0.01, and ****p < 0.0001, compared to control (CT) or Etoposide-treated cells (Eto) using Kruskal–Wallis followed by Dunn’s multiple comparison test (for **B**) and one-way ANOVA followed by Dunnett’s multiple comparison test (for **D**, **E**).

Considering the previous results and bearing in mind that the loss of cell proliferation is an important hallmark of cellular senescence, we proceeded to analyze the percentage of proliferative cells, following etoposide stimuli and a recovery period, in the presence of the essential oils. As expected, etoposide treatment resulted in a decrease in Ki67-positive cells, indicating a reduction in cell proliferation (Figures 3A,B). However, when the essential oils were added (200 μg/mL for *L. pedunculata* and 6.25 μg/mL for *L. stoechas* subsp. *luisieri*), during the recovery phase, the percentage of Ki67-positive cells was significantly increased, thus suggesting that these essential oils might attenuate senescence by either killing senescent cells (senolytic effect), or by modulating the senescence severity by modulating senescence-related pathways (senomorphic effect) (Figures 3A,B).

#### 3.4.2 Effect on senescence-associated β-galactosidase activity

In addition to cell proliferation, etoposide also led to a significant increase in SA-β-galactosidase staining, as expected, with cells presenting an evident increased size (Figures 3A,C). Importantly, both oils were able to decrease SA-β-galactosidase activity as shown by the decreased number of stained cells (Figures 3C–E). A noteworthy effect was observed for *L. stoechas* subsp. *luisieri* essential oil that induced a significant decrease (∼17%) at a very low concentration (6.25 μg/mL; Figures 3C,E), comparable to the reduction achieved with *L. pedunculata* at 200 μg/mL (Figures 3C,D).

#### 3.4.3 Nuclear accumulation of the phosphorylated form of γH2AX

**FIGURE 4:**
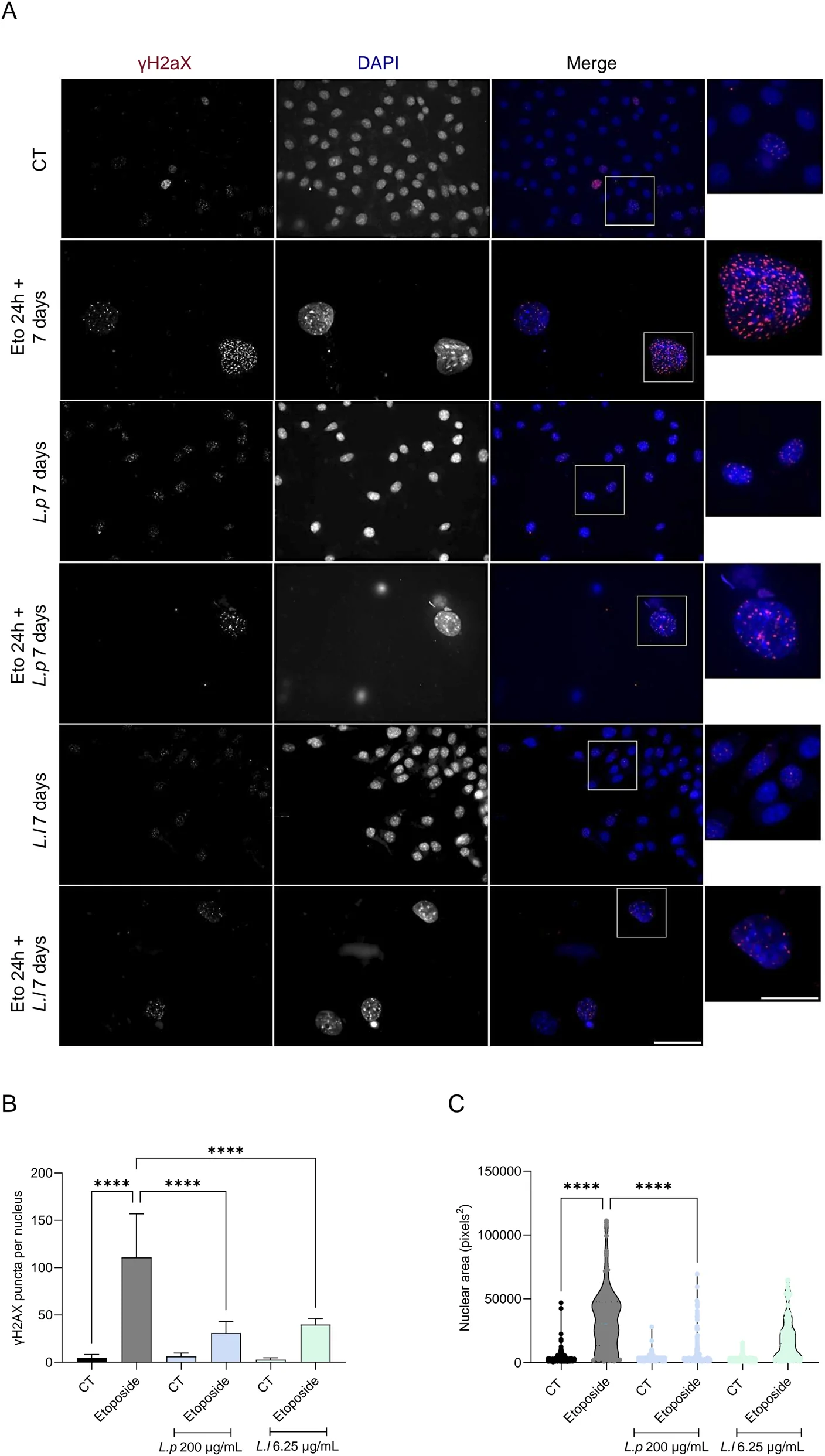
Effect of *Lavandula pedunculata* and *Lavandula stoechas* subsp. *luisieri* essential oils on γH2AX nuclear accumulation induced by etoposide. **(A)** Representative confocal images of NIH/3T3 cells treated with etoposide for 24 h followed by 7 days of recovery in etoposide-free medium in the presence or absence of the essential oils, followed by immunofluorescence for γH2AX (Alexa Fluor 647). Scale bar: 100 nm, 50 nm (Zoom-ins). **(B)** γH2AX puncta per nucleus. **(C)** Nuclear areas shown as pixels^2^. Results show the mean ± SEM (n = 3–4, minimum of 100 cells/n). ****p < 0.0001, compared to control (CT) or etoposide-treated (Eto) cells assessed by two-way ANOVA followed by Dunnett’s multiple comparison test (for **B**) and Kruskal–Wallis followed by Dunn’s multiple comparison test (for **C**).

Bearing in mind that etoposide induced an accumulation of γH2AX puncta in cell nucleus, after 7 days of recovery (Figures 2C,E), consistent with the accumulation of DNA double-strand breaks, we aimed to assess whether the presence of the essential oils could reduce this effect. Indeed, our results show that both essential oils were able to significantly decrease γH2AX puncta (Figures 4A,B). In addition, a decrease in nuclear area was evident, corroborating the previous results and suggesting that the nuclei may be reverting toward a proliferative state. In this case, the results were more significant for *L. pedunculata* (Figure 4C).

#### 3.4.4 Effect on p53/p21 signaling pathway

**FIGURE 5:**
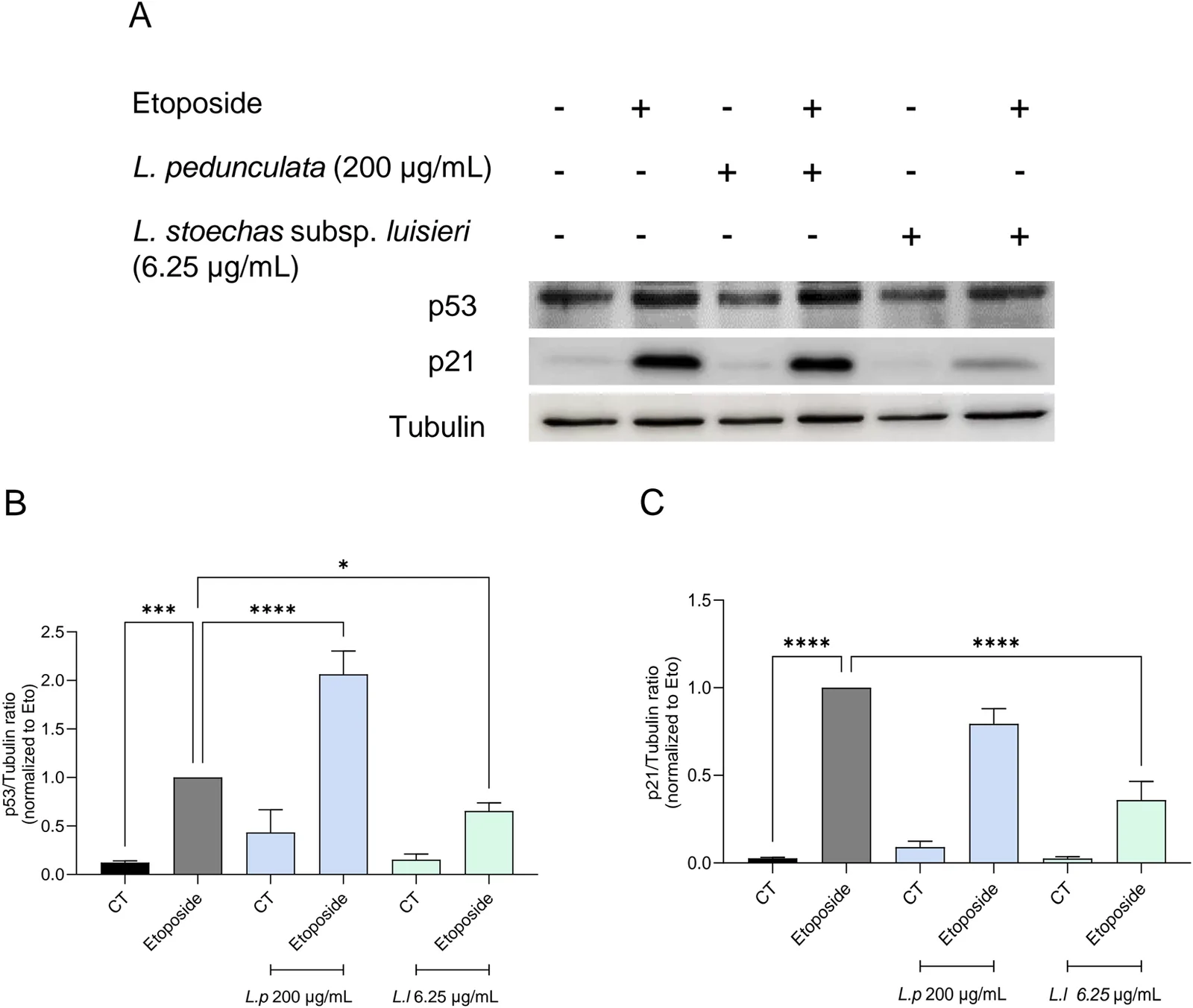
Effect of *Lavandula pedunculata* and *Lavandula stoechas* subsp. *luisieri* essential oils on p53/p21 signaling pathway. **(A)** Representative Western blot for p53 and p21 of NIH/3T3 cells treated with etoposide for 24 h followed by 7 days of recovery in etoposide-free medium in the presence or absence of the essential oils. Tubulin was used as loading control. **(B)** p53/Tubulin ratio. Values were normalized to etoposide-treated cells. **(C)** p21/Tubulin ratio. Values were normalized to etoposide-treated cells. Results show the mean ± SEM values (n = 4). *p < 0.05; ***p < 0.001 and ****p < 0.0001, compared to control (CT) or etoposide-treated (Eto) cells using one-way ANOVA followed by Holm-Šídák’s multiple comparison test (for **B**) or Dunnett’s multiple comparison test (for **C**).

Taking into account the promising effects observed in the previous experiments we proceeded to explore possible mechanisms of action underlying the anti-senescent potential of the essential oils. As DNA damage response is mediated by the p53/p21 signaling pathway (Di Leonardo et al., 1994; Campisi and d’Adda di Fagagna, 2007), we assessed the effects of the oils on the expression of these two proteins. As expected, etoposide-treated cells, following a 7-day recovery period, showed an increase in both protein expression levels (Figure 5). Interestingly, in the presence of *L. stoechas* subsp. *luisieri* essential oil, a significant decrease in the expression levels of both proteins was observed (Figures 5A–C), thus corroborating the anti-senescent results observed in the previous experiments. In contrast, *L. pedunculata* essential oil was only able to decrease the levels of p21 although no statistical significance was attained (Figures 5A–D).

#### 3.4.5 Ultrastructural alterations

**FIGURE 6:**
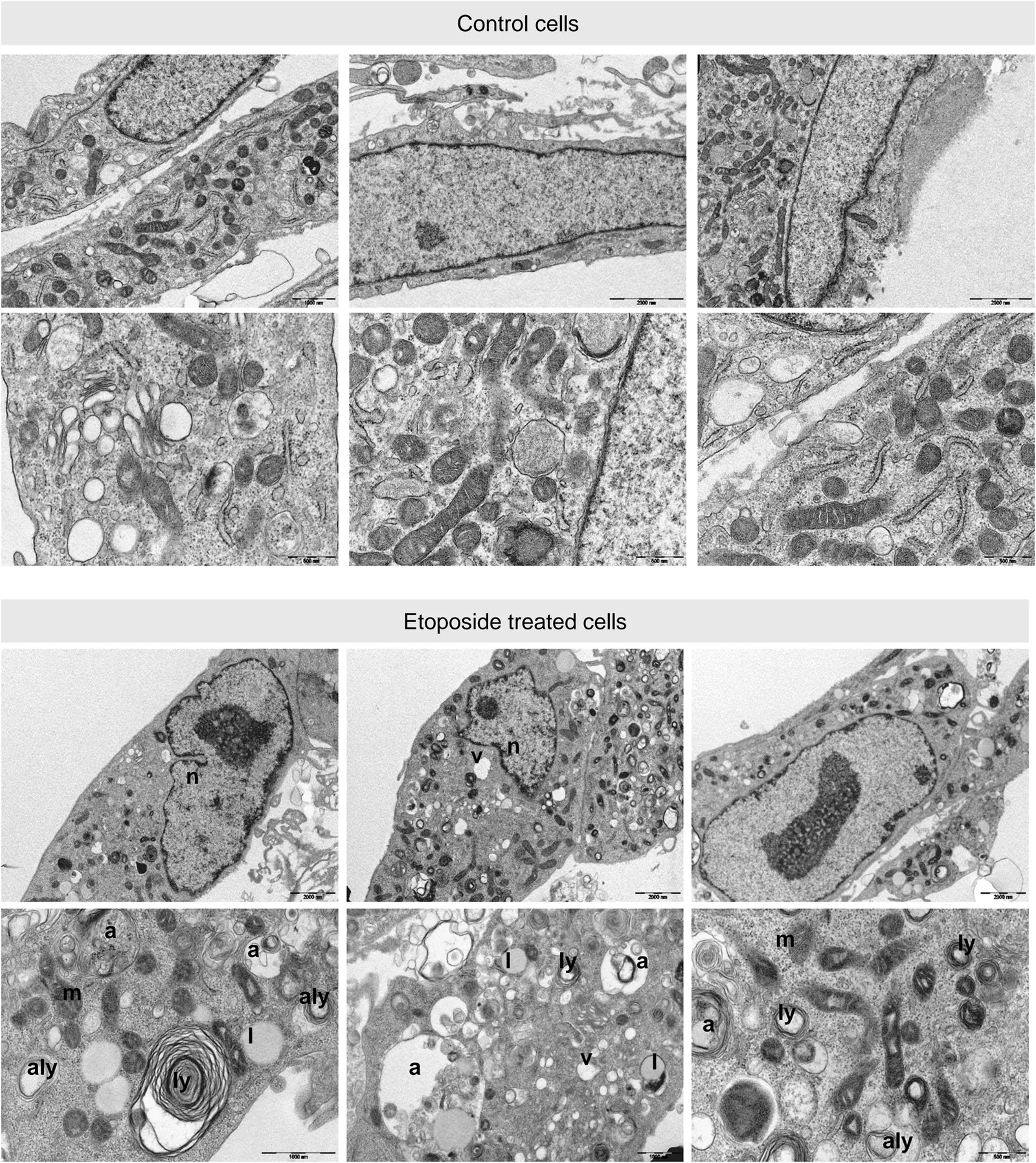
Ultrastructural modifications induced by etoposide. Representative TEM images showing evident ultrastructural alterations in NIH/3T3 cells following etoposide-induced senescence. Observed alterations include irregular shaped nuclei containing multiple invaginations and evident chromatin condensation (n), swollen mitochondria (m), and increased number of vacuoles (v), lidpid droplets (l), lysosomes (ly) and autophagic structures [autophagosomes (a) and autolysosomes (aly)]. Scale bars: 500 nm, 1,000 nm and 2,000 nm.

**FIGURE 7:**
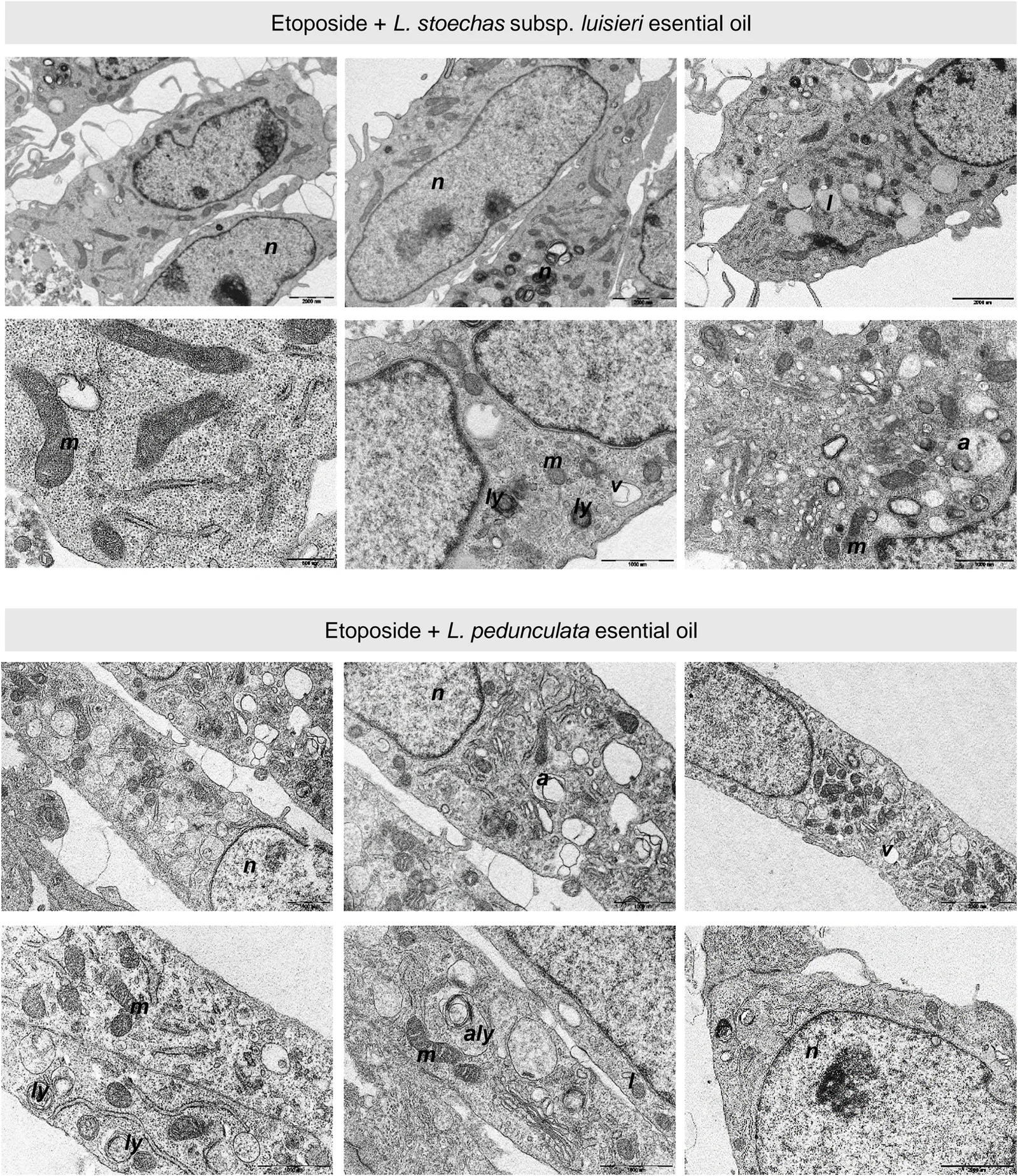
Effect of *Lavandula pedunculata* and *Lavandula stoechas* subsp. *luisieri* essential oil on cellular ultrastructure. Representative TEM images showing cellular ultrastructure following etoposide-induced senescence and a recovery period in the present of *Lavandula pedunculata* essential oil (200 μg/mL) or *Lavandula stoechas* subsp. *luisieri* (6.25 μg/mL) essential oil. Nuclei display a typical size and less invaginations (*n*); mitochondria show a normal morphology with defined cristae (*m*), and vacuolization (*v*), lidpid droplets (*l*), lysosomes (*ly*) and autophagic structures [autophagosomes (*a*) and autolysosomes (*aly*)] appear less prominent. Scale bars: 500 nm, 1,000 nm and 2,000 nm.

Transmission Electron Microscopy (TEM) analysis was used to assess the main ultrastructural alterations characteristic of cellular senescence, enabling a high-resolution visualization of organelle response to essential oil treatment. Following etoposide treatment, the typical features of cellular senescence were observed, namely, enlarged and irregular nuclei with invaginations and chromatin condensation, swollen mitochondria, increased vacuolization, accumulation of lipid droplets and lysosomes and increased autophagic structures (Figure 6). Following essential oil-treatment, cells presented an ultrastructure resembling healthy cells. Noteworthy, nuclei presented a typical size with less invaginations and chromatin condensation; mitochondria showed a normal morphology with defined cristae, some vacuolization persisted but in a much lesser extent, lipid droplets and lysosomes were less visible and autophagic structures were substantially diminished (Figure 7), indicating a restoration toward normal cell function.

#### 3.4.6 Senescence-associated secretory phenotype

**FIGURE 8:**
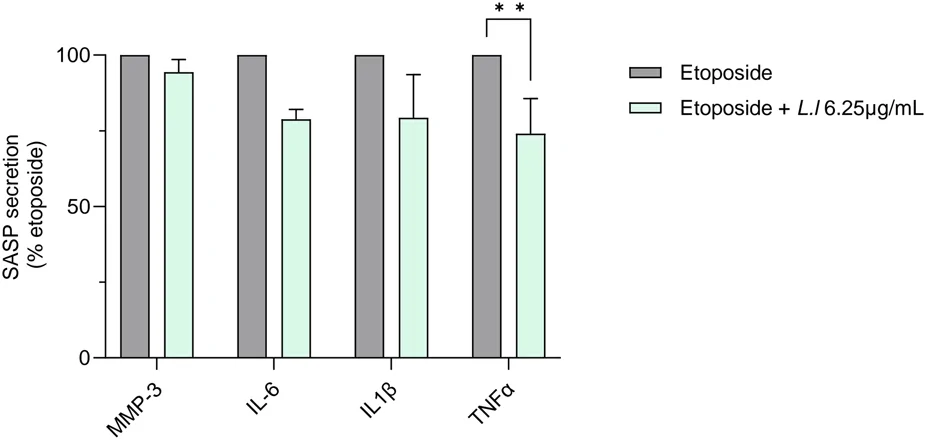
Effect of *Lavandula stoechas* subsp. *luisieri* essential oil on senescence-associated secretory phenotype. Secreted MMP-3, IL-6, IL-1β and TNF-α by NIH/3T3 cells treated with etoposide for 24 h followed by 7 days of recovery in etoposide-free medium in the presence or absence of *Lavandula stoechas* subsp. *luisieri* essential oil (6.25 μg/mL). Values were normalized to etoposide-treated cells. Results show the mean ± SEM of three independent experiments, **p < 0.01, compared to etoposide-treated cells using a mixed-effects analysis followed by Šídák’s multiple comparison test.

Senescence-associated secretory phenotype (SASP) comprises a variety of secreted chemokines, cytokines, proteins and growth factors that act in both an autocrine manner, reinforcing senescence within the cell, and in a paracrine way, inducing senescence in neighboring cells (Ohtani, 2022). Given the relevant effects shown for *L. stoechas* subsp. *luisieri* essential oil, we also assessed its impact on SASP secretion, namely, on the metalloproteinase MMP-3 and the pro-inflammatory cytokines TNF-α, IL-6, IL-1β, thus providing a more complete understanding of the oil’s senomorphic effect. As expected, etoposide-treated cells, following 7 days of recovery, secreted measurable amounts of the representative SASP components (Figure 8). The addition of the essential oil led to a decrease in the levels of these factors, although statistical significance was only achieved for TNF-α (Figure 8), thus suggesting a senomorphic potential of *L. stoechas* subsp. *luisieri* essential oil.

### 3.5 Effect of essential oils on key enzymes involved in skin aging

During aging several skin enzymes have increased activity leading to the visible signs of skin aging, namely, wrinkles, hyperpigmentation and overall loss of skin elasticity (Shin et al., 2023; Zolghadri et al., 2023). Therefore, the effect of the essential oils on relevant skin enzymes such as collagenase, tyrosinase, hyaluronidase, and elastase was assessed, providing mechanistic insights into how essential oils may preserve skin structure and function. Both oils were evaluated at different concentrations using an *in chemico* approach. Table 2 summarizes the main results, presenting the percentage of inhibition at the highest concentration tested. Overall, a modest inhibition on collagenase, tyrosinase, and hyaluronidase was observed for both essential oils, while for elastase no inhibition was detected. *L. stoechas* subsp. *luisieri* was the only oil that attained more than 50% inhibition of collagenase (IC_50_ = 674.9 μg/mL), suggesting a greater potential to modulate key enzymatic processes associated with skin aging and extracellular matrix degradation.

**TABLE 2:** Enzymatic modulation of *Lavandula* spp. essential oils.

Essential oil	Collagenase (%)	Hyaluronidase (%)	Tyrosinase (%)
*Lavandula pedunculata*	41.2	31.2	33
*Lavandula stoechas* subsp. *luisieri*	58.1	34.2	9

## 4 Discussion

The genus *Lavandula* is an important source of essential oils highly valuable in the cosmetic and perfumery industries (Cavanagh and Wilkinson, 2005). However, among the 39 described species only four are commercially relevant (Upson and Andrews, 2004). In fact, most of the species remain unbranded and underexplored despite their huge potential. Some of these species are valued in traditional medicine and have scientific studies supporting their bioactive potential. This is the case of *L. pedunculata* and *L. stoechas* subsp. *luisieri,* two endemic species of the Iberian Peninsula. Over the years, these species have shown very promising antifungal and anti-inflammatory properties (Zuzarte et al., 2009; 2012; Zuzarte et al., 2022; Domingues et al., 2023). For *L. pedunculata* different chemotypes have been identified in Portugal, based on the predominance of three main compounds, namely, fenchone, 1,8-cineole and camphor. The samples collected in Serra da Malcata region and herein analyzed fall within the fenchone chemotype (Zuzarte et al., 2022). On the other hand, *L. stoechas* subsp. *luisieri* exhibits a unique chemical profile within the plant kingdom, characterized by the presence of necrodane derivatives (García-Vallejo et al., 1994; Sanz et al., 2004; Baldovini et al., 2005; González-Coloma et al., 2011; Zuzarte et al., 2012; Zuzarte et al., 2022), a class of compounds first identified in the secretion of the carrion beetle *Necrodes surinamensis* (Roach et al., 1990). The samples analyzed in the present study are particularly noteworthy as these necrodane derivatives attained 28.1% of the total oil composition. This particular chemical profile seems to underline the remarkable anti-senescent effects herein reported for *L. stoechas* subsp. *luisieri*, as significant protective effects were observed at very low concentrations (6.25 μg/mL). Indeed, our results show that the essential oil from this species was able to decrease both p53 and p21 proteins, thus suggesting a modulatory effect on the p53/p21 signaling pathway. Although the precise molecular targets of these constituents remain to be elucidated, their unique chemical architecture may contribute to the observed bioactivity through modulation of stress-responsive and cell cycle regulatory pathways. In particular, the concurrent downregulation of p53 and p21 suggests an effect on upstream signaling events involved in cellular stress responses and senescence induction, thereby warranting further mechanistic investigation. In this context, essential oils from *L. stoechas* subsp. *luisieri* have previously been associated with antioxidant and anti-inflammatory activities, which may further support a role for this distinctive chemical profile in modulating stress-responsive and inflammatory pathways that contribute to cellular senescence. Corroborating this finding was the evident decrease in senescence biomarkers, such as β-galactosidase activity, nuclear accumulation of the phosphorylated form of γH2AX and SASP release, after a 7-day recovery period, post senescence-induction. Furthermore, cell proliferation increased, and nuclear and cellular morphology, as well as cell ultrastructure returned to a normal state. It is well established that p21 mediates the senescence-associated secretory phenotype (SASP) secretion (Sturmlechner et al., 2021). Therefore, the reported lowering effect of *L. stoechas* subsp. *luisieri* essential oil on SASP, most notably TNF-α, might be attributed to its significant inhibition of p21 expression levels. Nevertheless, a more comprehensive evaluation of additional SASP components, together with an increased number of biological replicates, will be valuable to further substantiate and extend these observations, thereby providing a more detailed characterization of its anti-inflammatory and senescence-modulating potential. Importantly, SASP regulation is influenced by multiple interconnected factors. In fact, persistent DNA damage has been reported to promote SASP (Rodier et al., 2009). Therefore, the decrease in DNA damage foci noticed in the presence of *L. stoechas* subsp. *luisieri* essential oil might also play a role in decreasing SASP secretion. In addition, the nuclear factor kappa-light-chain-enhancer of activated B cells (NF-κB) binds to and accumulates in the chromatin of senescent cells, driving the elevated expression of senescence-associated and inflammatory genes (Chien et al., 2011), thereby promoting SASP secretion (Kang et al., 2015). Furthermore, it was shown that inhibiting or disrupting this pathway decreases growth arrest and the release of SASP components (Chien et al., 2011; Rovillain et al., 2011). *L. pedunculata* essential oil was also effective, although a higher concentration was needed to decrease the typical markers of cellular senescence. To the best of our knowledge, the present study is the first to report the anti-senescent properties of *Lavandula* essential oils, however some studies have reported these effects for non-volatile extracts (Zhao et al., 2015; Ha et al., 2019; Salem et al., 2020), further validating the potential of this genus as a source of senotherapeutics. Regarding isolated compounds, typically found in these essential oils, studies addressing anti-senescent effects are also scarce, with only one study reporting the anti-senescent potential of camphor. This compound was able to inhibit SA-β-gal activity in hydrogen peroxide-induced senescence and prevent UV-induced wrinkle formation (Tran et al., 2015), thus reinforcing its anti-aging potential. Nevertheless, caution is warranted, as camphor may cause contact dermatitis, skin irritation or systemic toxicity can occur (FDA, 2023). Furthermore, camphor is included on the European Commission´s list for allergens for cosmetic products (European Commission, 2023), further highlighting the need for careful formulation and safety assessment in topical applications.

Bearing in mind the anti-aging potential of *L. stoechas* subsp. *luisieri* essential oil, its putative effect on relevant skin enzymes was also assessed as an increase in these enzymes, namely, collagenase, hyaluronidase and elastase, is often observed in aged skin, leading to the formation of wrinkles and loss of skin elasticity (Shin et al., 2023). Furthermore, aged skin is often affected by hyperpigmentation due to increased activity of pigmented cells, particularly melanocytes, where tyrosinase is present (Yang et al., 2025). Overall, *L. stoechas* subsp. *luisieri* essential oil decreased the majority of enzymes activity, thereby demonstrating its potential to prevent or mitigate skin aging. While these findings are significant in demonstrating the ability of *L. stoechas* subsp. *luisieri* to inhibit key enzymes associated with the decline of skin health, some limitations should be considered. First the enzymes used in these assays are not of human origin, and enzyme kinetics can be species-dependent (Vinardell et al., 2024). Furthermore, essential oils are highly apolar whereas *in chemico* assays often employ water-based solutions, which may underestimate their true activity. Therefore, although *in chemico* assays are valuable for initial screening purposes, more physiologically relevant models are required to confirm these effects, which may not result from direct enzyme inhibition but rather from the modulation of cellular pathways involved in the regulation of enzyme expression, secretion, or activation.

Importantly, studies addressing the effect of *Lavandula* spp. extracts on skin enzymes are scarce. One study reported that the essential oil from *L. angustifolia* is able to increase procollagen synthesis in human skin fibroblasts (Andrys et al., 2018). Another study demonstrated that ethanolic and aqueous extracts from *Lavandula officinalis* inhibit the activity of elastase, collagenase and tyrosinase (Salem et al., 2020). Regarding isolated compounds, it was reported that camphor was able to inhibit elastase activity in dermal fibroblasts while increasing the levels of collagen, thus suggesting that it might either inhibit collagenase activity or promote collagen synthesis (Tran et al., 2015). Another study reported that fenchone and camphor were able to inhibit the activity of tyrosinase and elastase (El Omari et al., 2023).

Overall, our results demonstrate a strong anti-senescent potential, particularly for *L. stoechas* subsp. *luisieri* essential oil, as supported by multiple experimental approaches. Although the present findings provide robust evidence of the anti-senescent effects of the tested essential oils in a well-established fibroblast model, their validation in primary human dermal fibroblasts and other relevant human skin cell models will be important to further assess their translational relevance and potential application in skin health and cosmeceutical development. This species exhibited activity at very low concentrations, while containing a relatively low concentration of camphor, which may reduce the risk of irritation or toxicity. These findings position *L. stoechas* subsp. *luisieri* as a promising candidate for cosmeceutical applications, particularly in topical formulations such as creams or gels that are easy to apply and can deliver active compounds effectively to the skin. Beyond its bioactivity, this essential oil offers a natural sensory appeal due to its fragrance and lipophilic nature which may allow deeper penetration of active compounds, further enhancing efficacy. However, several challenges must be addressed as essential oil composition can vary due to geographic, seasonal, or extraction factors, which may affect reproducibility and efficacy in commercial products. In addition, safety-related aspects, including skin sensitization, irritation potential, formulation stability, and standardization, require careful evaluation prior to any topical application. In particular, dedicated skin sensitization and irritation studies will be essential to ensure the safety of any potential cosmeceutical formulation containing *L. stoechas* subsp. *luisieri* essential oil. Finally, to ensure sustainability and protect natural populations, wild collection should be avoided, with production relying on propagated plants, for example, through micropropagation, to maintain consistent quality and supply. Future studies using cellular and *in vivo* models are also crucial to optimize formulations, validate efficacy, and ensure safe topical use, bridging the gap between experimental findings and practical cosmeceutical applications.

## 5 Conclusion

The present study reports for the first time the anti-senescent potential of two *Lavandula* species endemic to the Iberian Peninsula. Both essential oils showed potent anti-senescent potential, with *L. stoechas* subsp. *luisieri* standing out due to its effective inhibitory effect at a very low concentration. This species is also of interest due to the unique chemical composition of its essential oil, since necrodane-type compounds are not common in the plant kingdom.

Collectively, the data support the ability of the essential oil to modulate key hallmarks of cellular senescence. Nevertheless, whether these effects reflect suppression of the senescent phenotype (senomorphic activity), selective elimination of senescent cells (senolytic activity), or a combination of both remains to be established. Moreover, further studies using more physiologically relevant systems are needed to elucidate these effects. In combination with previously reported anti-inflammatory and anti-fungal effects for these essential oils, this work validates their traditional relevance and highlights their potential to be used in cosmeceutical formulations, aiming at improving the overall skin health.

## Data Availability

The raw data supporting the conclusions of this article will be made available by the authors, without undue reservation.
